# Supplementary material for: Detection of epistasis between ACTN3 and SNAP-25 with an insight towards gymnastic aptitude identification
Source: PLoS One. 2020 Aug 31;15(8):e0237808. doi: 10.1371/journal.pone.0237808 (PMC7458280; doi:10.1371/journal.pone.0237808)
Supplement: Attachment — Submitted filename: recommendation.doc [file pone.0237808.s005.doc]

In this study, the authors performed an analysis of gene-gene interaction of seven PEPs (rs1815739, rs8192678, rs4253778, rs6265, rs5443, rs1076560, rs362584) with gymnastic aptitude in a case (gymnasts)-control (sedentary individuals) setting. The rs1815739 x rs362584 epistatic regression model exhibited a good fit to the data achieving a significant improvement in sportsmen identification over naïve guessing. We have some doubt：

1. As we know that only one allele could not represent a gene. If you want to study the interaction of these 7 genes（ ACTN3，PPARGC1A，PPARα，BDNF，AS GNB3， DRD2， SNAP-25）, you should include all tag SNPs.
2. Please give the statistical power of this study.
